# Validity of FFQ Estimates of Total Sugars, Added Sugars, Sucrose and Fructose Compared to Repeated 24-h Recalls in Adventist Health Study-2 Participants

**DOI:** 10.3390/nu13114152

**Published:** 2021-11-19

**Authors:** Mericarmen Peralta, Celine Heskey, David Shavlik, Synnove Knutsen, Andrew Mashchak, Karen Jaceldo-Siegl, Gary E. Fraser, Michael J. Orlich

**Affiliations:** School of Public Health, Loma Linda University, Loma Linda, CA 92350, USA; cheskey@llu.edu (C.H.); dshavlik@llu.edu (D.S.); sknutsen@llu.edu (S.K.); amashchak@llu.edu (A.M.); kjaceldo@llu.edu (K.J.-S.); gfraser@llu.edu (G.E.F.); morlich@llu.edu (M.J.O.)

**Keywords:** sugar intake, validation studies, quantitative food frequency questionnaire

## Abstract

Sugar intake is a potentially important aspect of diet which has not previously been validated in the Adventist Health Study-2 (AHS-2). We sought to validate the food frequency questionnaire (FFQ) measurement of total sugars, added sugars, sucrose, and fructose against multiple 24-h dietary recalls (recalls) in AHS-2 participants. Food consumption data from a self-administered FFQ and six recalls from 904 participants were combined with nutrient profile data to estimate daily sugar intake. Validity was evaluated among all participants and by race. FFQ and recall means were compared and correlation coefficients (Spearman’s, energy-adjusted log-transformed Pearson’s, deattenuated Pearson’s) were calculated. Mean total energy, total sugars, and fructose intake were higher in the FFQ, whereas added sugars and sucrose were higher in recalls. The energy-adjusted (log-transformed) deattenuated correlations among all participants were: total sugars (r = 0.42, 95% CI 0.32–0.52), added sugars (r = 0.50, 95% CI 0.36–0.59), sucrose (r = 0.32, 95% CI 0.23–0.42), and fructose (r = 0.50, 95% CI 0.40–0.59). We observed moderate validity for added sugars and fructose and low-moderate validity for total sugars and sucrose measured by the AHS-2 FFQ in this population. Dietary sugar estimates from this FFQ may be useful in assessing possible associations of sugars intake with health outcomes.

## 1. Introduction

The health effects of sugar consumption have been a topic of considerable interest and controversy. Epidemiologic evidence has linked over-consumption of added sugars to deleterious health conditions [1,2], such as obesity [3,4,5], cardiovascular disease [6,7,8,9], and potentially cancer [10]. Increasing concern by United States regulatory agencies is reflected in recent guideline changes to nutritional food labels requiring added sugars to be included on all labels by 2021 [11].

Tools such as self-reported food frequency questionnaires (FFQs) utilized to estimate intake of dietary sugars are susceptible to measurement errors [12]. Yet FFQs remain the most utilized form of collecting dietary data in large cohort studies [13,14,15,16]. Validation studies comparing FFQs to reference methods (typically multiple food recalls or records) are commonly used to quantitatively estimate accuracy. Consequently, validated FFQs can serve as primary assessment of dietary intake for studies of association diet to disease.

The Adventist Health Study-2 (AHS-2) is a prospective cohort study of Adventists in the USA and Canada with a diversity of dietary patterns including many vegetarians [17]. The FFQ of the AHS-2 has previously been validated for a wide range of nutrients, including non-fiber carbohydrates, and foods [18,19]. However, measurement of dietary sugars has not been validated in AHS-2. The purpose of this paper is to validate the AHS-2 FFQ for the assessment of sugars intake within the AHS-2 population (compared to multiple dietary recalls).

## 2. Materials and Methods

### 2.1. Study Population

The AHS-2 is a cohort study of approximately 96,000 adult male and female Seventh-Day Adventist participants in North America, recruited from 2002 to 2007 [15]. The study protocol was approved by the IRB of Loma Linda University, and signed consent was obtained prior to subject enrollment [15]. A calibration sub-study of AHS-2 was conducted using 1085 randomly selected subjects of the larger cohort study. Black participants were over sampled to allow for race-specific validation and calibration with adequate power. The current analysis included participants who completed the FFQ from the calibration study and responded to the 24-h recalls (*n* = 1085). The following exclusions were applied: participants with a missing 24-h recall within the first synthetic week (i.e., first set of 3 recalls) (*n* = 81), participants with an FFQ missing a large number of responses (missing greater than 69 of the 204 food items) (*n* = 88), and participants with estimated energy intakes of less than 500 kcal (*n* = 4) or greater than 4500 kcal (*n* = 8) (defined by cohort investigators as extreme daily energy intake values and standardly excluded in diet-disease analyses in this cohort), leaving an analytic sample of 904 participants. Twenty-one participants had unknown race, so 883 participants were included in race-specific analyses. Forty-two subjects were not able to be included in deattenuation analyses due to an insufficient number of completed recalls (missing the second set of 3 recalls), leaving 862 persons in these analyses (843 with known race).

### 2.2. Calibration Study Design

The calibration study began approximately 1–2 years after the baseline questionnaire was administered. Within the 9–12-month calibration study period, two sets of three 24-h dietary recalls were administered approximately 6 months apart, and an FFQ was administered between these two sets of recalls [18]. Each set of three recalls was administered within a 2-month timeframe.

### 2.3. Dietary Assessment

#### 2.3.1. FFQ

All AHS-2 participants completed a baseline diet and lifestyle questionnaire designed to measure lifestyle, physical activity, and medical history, including an FFQ (hereafter referred to as the baseline FFQ). Calibration study subjects completed another self-administered quantitative FFQ (hereafter referred to simply as the FFQ unless baseline FFQ is specified; used for all validity analyses) assessing dietary intake over the previous one year. This FFQ included the same foods as the baseline FFQ. The complete FFQ consisted of 204 foods with questions pertaining to frequency of consumption and portion sizes (standard, 1/2 of standard or less, and 1 1/2 or more). Standard serving sizes were given for each food question using pictures and familiar units (for example tablespoons or cups) to assist in estimating portion sizes. Write-in options for food items were available within each section. Frequency options for most foods included never, 1–3 times per month, once per week, 2–4 times per week, 5–6 times per week, 1 per day and, and 2 plus times per day. In some cases, subjects were asked to consider additional questions about food preparation.

#### 2.3.2. Nutrient Calculations for FFQ

The Nutrition Data System for Research (NDSR v5.0.) (of the Nutrition Coordinating Center at the University of Minnesota) was used to estimate the nutrient content of foods. Food consumption data gathered from the FFQ combined with the nutrient profile data from NDSR was used to estimate daily nutrient consumption for each AHS-2 subject. The product-sum method of calculating the nutrient intake was used: the sum of frequency × portion size × amount of the nutrient per serving across all foods [20].

The intake of four sugars was estimated: Total sugars (the total of all free mono- and disaccharides in foods, including those naturally occurring and those added); added sugars (sugars added during processing); and sucrose (a disaccharide composed of fructose and glucose) and fructose (a monosaccharide), which are often consumed in larger quantities as added sugars. Total sugars, added sugars, fructose, and sucrose were all available as outputs from NDSR.

Most write-in food questions were previously transcribed for all participants, and nutrients from these items were calculated in similar fashion to foods listed in the questionnaire. There were several write-in food items not previously transcribed (other juices, canned fruits, and hot drinks). For this analysis, these items were transcribed for a random sample of participants: 4712 of the 55,436 total reported other juices (8.5%), 6196 of the 63,233 total other canned fruits (9.8%), and 7845 of the 54,484 total other hot drinks (14.4%).

Responses from these samples were then analyzed and used to create a generic recipe for each write-in item. The generic recipe was created as follows: For each item relevant to that food category, all the foods transcribed representing more than three percent of the written-in responses at any consumption frequency were weighted by frequency and summed to produce a mixed food component, or recipe. This mixed component was assigned to each subject with a written-entry for that item (see Supplementary Tables S1–S3 for an example of the components created for other fruit juice, canned fruits, and hot drinks). Nutrient calculation for these mixed components produced from written-in food items then proceeded in a similar fashion as for foods already listed in the questionnaire. The most common written-in items were: peaches, apple or applesauce, and pineapple for other canned fruit; green tea, herbal tea, coffee substitute, and hot chocolate mixture for other hot drink; cranberry drink, grape juice, and mixed fruit juice for other fruit juice.

#### 2.3.3. Dietary Recalls

Data collection for 24-h recalls consisted of unannounced telephone calls by trained research dietitians using a standard probe and multiple-pass method [18]. Dietitians questioned participants on foods, beverages, and supplements consumed during the previous 24 h. NDSR version 5.0 was again used to determine nutrient (i.e., total sugars, added sugars, sucrose, and fructose) composition of foods. The sum of nutrient intake across all foods from each 24-h recall represented subject nutrient intake for that day.

Data from each set of three 24-h recalls (a Saturday, a Sunday, and a weekday) resulted in the production of two synthetic weeks of nutrient intake estimate. For each synthetic week, an average daily intake was calculated (formula below) [18].

(1) $$\begin{array}{l} \mathrm{Average}\;\mathrm{daily}\;\mathrm{intake}\;\mathrm{for}\;\mathrm{synthetic}\;\mathrm{week}\\ =\frac{\left({\mathrm{Saturday}}_{\mathrm{Intake}}+{\mathrm{Sunday}}_{\mathrm{Intake}}+\left(5\times {\mathrm{Weekday}}_{\mathrm{Intake}}\;\right)\right)}{7} \end{array}$$

The average daily intakes for the two synthetic weeks were then averaged to produce an overall average daily nutrient intake, and used separately for deattenuation of within-subject variation. If a subject had an incomplete week 2, then values for week 2 were excluded and outcomes for week 1 represented the value for the overall average daily nutrient intake. As a result, subjects with only one synthetic week were excluded when calculating deattenuated correlations.

### 2.4. Statistical Analysis

Independent *t*-tests and chi-squared tests were used to compare participant characteristics by race. Means, standard deviations, and selected percentiles of estimated daily intake of dietary sugars were calculated for all subjects and stratified by race for 24-h recalls and FFQ.

Validity correlations (comparing daily intakes from FFQ and 24-h recalls) were calculated for each nutrient using untransformed non-energy-adjusted values (non-parametric Spearman correlation) and using log-transformed energy-adjusted values (parametric Pearson correlation). As they were strongly right skewed, nutrient values for Pearson correlation were log-transformed: log(x + 1) of energy adjusted FFQ and log(x+1) of average 24-h dietary sugar intake (which were approximately normally distributed after transformation). Dietary sugar intake values were energy-adjusted using the residual method, adding the residual of the predicted nutrient intake for each individual to the mean intake [21,22]. Since previous validation of multiple nutrient intake using the AHS-2 calibration data revealed significant differences in validity by race and not by gender, race-specific analysis was applied in this study [19].

Cross classification tables by quintile were examined to evaluate the extent of misclassification. The percent of subjects that were classified in the same quintile (exact) or within one quintile (adjacent) as well as indicating gross misclassification (opposite extreme quintiles) was assessed.

Large within-person variations in diet tend to attenuate and make less significant correlations between the test (FFQ) and the reference method (24-h recalls). Therefore, deattenuation of the correlation coefficients using the ratio of within-person to between-person variation calculated from week one and week two of nutrient intake from the 24-h recalls was performed, [19] per the formulas displayed below:

(2) $$r_{t}=r_{O}\;\sqrt{1+\frac{\lambda_{x}}{2}}$$

where *r_t_* = true correlation, *r_O_* = observed correlation, *λ_x_* = ratio of within and between person variance for *x*, and 2 = number of replicates per person [20]. Since between-person variance is equal to the total variance minus the average within-person variance divided by the replicates per person [23],

(3) $$Var_{T}-\frac{Var_{W}}{2}$$

the deattenuated correlation coefficient was determined by using the following formula:

(4) $$r_{c}=r_{O}\sqrt{1+\frac{1}{2}\left(\frac{Var_{W}}{Var_{T}-\frac{Var_{W}}{2}}\right)}$$

where *r_c_* = deattenuated correlation coefficient, *r_o_* = uncorrected correlation, 2 = number of replicate measures per person (i.e., synthetic weeks), *Var_T_* = variance of all 24-h recall measures, and *Var_W_* = pooled estimate (mean) of within-subject variances.

Confidence intervals for corrected correlation coefficients were calculated using bootstrap resampling and the bias corrected accelerated (BCα) method performed in Rstudio [24,25]. All previous analysis including quintile cross classification tables and scatter plots for each nutrient was examined using SAS statistical software package version 9.4 (SAS System for Windows. Copyright © 2016 SAS Institute Inc., Cary, NC, USA.) [26].

## 3. Results

In total, there were 904 participants in this study. Table 1 presents descriptive statistics for all participants and separately for Black (*N* = 376) and non-Black (*N* = 507) participants (21 participants were missing identified race). *p* values are for statistical tests of significant differences by race. Compared to non-Blacks, Black participants were more likely female, were on average younger, were less likely to be vegetarian, were more likely to be obese (BMI 30+), and had higher average BMI.

Mean intake of total energy, total sugars, and fructose (Table 2) were higher in the FFQ than in the 24-h recalls, whereas added sugars and sucrose were higher in the 24-h recalls compared to the FFQ. These differences were reflected across most percentiles. Scatter plots representing the correlations between FFQ and recall measurements for each sugar are provided as Supplementary Figure S1.

The average total energy, total sugars, and fructose intake was higher when measured by FFQ compared to 24-h recalls among Black participants (Table 3). Similarly the non- Black population averaged higher total energy and fructose intake in FFQs vs. 24-h recalls. However, FFQ estimated total sugar, such as added sugars and sucrose intake, among non-Black respondents was lower when compared to mean 24-h recalls.

Spearman’s correlations (Table 4) for the (non-energy adjusted) values of FFQ and 24-h recalls revealed weak positive correlations for all sugars: r = 0.19 (for total sugars), 0.30 (for added sugars), 0.16 (for sucrose), and 0.30 (for fructose). The energy adjusted (log-transformed) Pearson’s correlations (of FFQ and average 24-h recall) were more strongly correlated for all sugars compared to the non-energy adjusted Spearman’s correlations, but remained modest: total sugars (r = 0.32), added sugars (r = 0.33), sucrose (r = 0.21), and fructose (r = 0.40). This was also true among both Black and non-Black participants. The energy-adjusted validity correlations for non-Black participants were greater than those for Black participants except for sucrose.

Deattenuation (for within person variation in 24-h recalls) strengthened the validity correlations for all nutrients. Deattenuated correlations (Table 5) were as follows: total sugars (r = 0.40, 95% CI 0.31–0.50), added sugars (r = 0.48, 95% CI 0.38–0.58), sucrose (r = 0.32, 95% CI 0.21–0.42), and fructose (r = 0.50, 95% CI 0.40–0.58). Deattenuated correlations for Black participants remained notably lower for total sugars, somewhat lower for added sugars and fructose, but somewhat higher for sucrose.

When dietary sugars intake was categorized into quintiles of intake (Supplementary Table S4), cross-classification across all dietary sugars showed that between 23.1 and 28.5% of the subjects were correctly classified in exact agreement between the FFQ and the average 24-h recalls. Adjacent agreement (1 quintile deviation) ranged from 32.9 to 36.4%. Agreement within one quintile (including exact agreement) ranged from 56.7 to 61.5%. Gross misclassification into opposite extreme quintiles for sugars intake was 3.1–6.7%. Energy adjustment tended to improve cross classification agreements. Differences in race were small, yet Black participants demonstrated a higher degree of misclassification into extreme quintiles and lower percentage of agreement within one quintile compared to non-Black participants.

## 4. Discussion

In this study, dietary sugars intake from the AHS-2 FFQ was assessed against multiple 24-h recalls. The deattenuated correlations for all participants were: total sugars r = 0.40 (95% CI, 0.31–0.50), added sugars r = 0.48 (0.38–0.58), sucrose r = 0.32 (0.21–0.42), and fructose r = 0.50 (0.41–0.58). These findings indicate that the AHS-2 FFQ provides estimates of moderate validity for dietary sugars. These results are similar to those previously reported for many other nutrients in AHS-2 [27,28,29,30]. Such moderate validity is not atypical of FFQs in epidemiological studies, where correlations of 0.5 to 0.7 are often considered desirable [20]. We now briefly compare the present validity correlations for sugars with those published from other cohorts.

For total sugars intake, compared to r = 0.40 in AHS-2, the European Prospective Investigation into Cancer (EPIC) study found a deattenuated correlation coefficient of r = 0.73 comparing a 7-day food diary to the FFQ [31]. The Malmo Food Study reported a correlation for total sugars of r = 0.67 when using an 18-day food diary as reference [32]. Validity for total sugars in The Japan Public Health Center Based Prospective Study (JPHC) was r = 0.52 for males and r = 0.36 for females comparing a 7-consecutive-day diet record and FFQ [28].

For added sugars, compared to r = 0.50 in AHS-2, the Norwegian Women and Cancer Study FFQ compared to four 24-h dietary recalls during a year had an energy-adjusted correlation of r = 0.65 (95% CI, 0.47–0.82) [33]. The Inter99 study, among a Danish population, reported added sugars correlations for men of r = 0.66 and women of r = 0.51 when comparing the FFQ to a 28-day diet history [34].

For sucrose, compared to r = 0.32 in AHS-2, the Nurses’ Health Study (NHS) reported an energy adjusted correlation of r = 0.54 comparing the FFQ to four one-week diet records. In JPHC, validity for sucrose was r = 0.46 for males and r = 0.38 for females when comparing the FFQ with 7 consecutive dietary records over 4 seasons [28].

For fructose, compared to r = 0.50 in AHS-2, validity in JPHC study was r = 0.54 for males and r = 0.31 for females. In general, then, the validity of the AHS-2 FFQ for measuring dietary sugars was similar to that achieved in some studies (e.g., JPHC), but not as high as that achieved in others (e.g., EPIC).

In this analysis, the average intakes derived from the FFQ were higher than those from 24-h recalls for total energy, total sugars, and fructose. For total energy, this is consistent with previous findings in the AHS-2 population [18] as well as many other studies [33,35]. However, this was not true of added sugars and sucrose, which were under-estimated by the FFQ compared to recalls. One reason for this underestimation by FFQ (compared to recalls) may be greater desirability bias (i.e., underreporting due to fear of judgement or desire to report healthier behaviors) for sucrose and added sugars since these nutrients are more related to dessert items and sweetened beverages. Such a bias might be stronger when the participant is asked about usual dietary intake rather than items eaten on a particular day. Another likely reason could be that there were not enough questions on the FFQ about foods high in added sugars and sucrose. For example, the FFQ lacked specific questions about chocolate, candy, table sugar, and honey. The higher FFQ estimates for total sugars and fructose may simply reflect the higher estimated total energy intakes of the FFQ. The FFQ may also have some overreporting of foods high in naturally occurring sugars, such as fruits.

Unlike other cohorts, this study assesses the FFQ at a race specific level. The AHS-2 intentionally recruited Black participants, as part of an under-studied community subject to health disparities. Results of a previous pilot study contributed to the design of the FFQ to include items commonly eaten among Black populations, including those of Caribbean origin or from the southern region of the United States [36]. Black participants were also over-sampled in the validation study.

Despite these efforts, the deattenuated energy-adjusted correlations were generally lower among Black participants (except for sucrose), for example r = 0.36 for Black vs. r = 0.55 for non-Black participants for total sugars. This suggests that future efforts would be warranted to optimize FFQ design with respect to Black participants.

The mean total sugars, added sugars, and sucrose intake observed in 24-h recalls in our population were lower than those reported from NHANES (total sugars 108 g, and added sugars 54.8 g in males aged 55+) [37,38] even among vegetarians (average added sugar intake in NHANES vegetarians 63.2 ± 4.4 g vs. AHS-2 vegetarians 49.91 ± 30.40 g see Supplementary Table S5) [39]. Such intake differences may be reflective of low total dietary energy intake. In this study, total energy intake was considerably lower than the typical American diet of approximately 2200 calorie intake per day [38,40], both by FFQ (total energy 1811.30 kcal/day) and by 24-h recall (total energy 1563.66 kcal/day) [38]. AHS-2 sugar intakes were also slightly lower compared to nationally representative dietary surveys from other countries: total sugars intake in Sweden (87.8 g) and United Kingdom (95.1 g), added sugars intake in Denmark (48 g) and Iceland (58.8 g), and sucrose intake in Netherlands (52 g in males) and Finland (53 g in males) [37,41]. The estimated average daily added sugar intake of AHS-2 participants is 45 g or approximately 12% of the average total daily calories consumed and relatively close to the <10% of daily total caloric intake recommended by the US Dietary Guidelines and the World Health Organization [11]. Since these differences are from 24-h recalls, and thus not attributable to FFQ design, it is likely that these differences arise from the composition (e.g., older average age) and characteristics (e.g., health-consciousness, tendency to healthy vegetarianism) of the AHS-2 population. The average age of subjects in this sub-study was 56 years old at enrollment. Older adults have lower daily energy needs and may consume less added sugars compared to younger adults and adolescents [37]. Alcohol is estimated to contribute approximately 3.7% [42] to US dietary energy intake, so very low alcohol intake in the Adventist population may contribute to a reduced total caloric intake. Participants of the AHS-2 cohort are a religiously-motived health-conscious group and may thus avoid excess intake of sugars.

This study has several strengths. Six 24-h recalls (rather than one or two) were used as the reference for evaluating dietary sugars intake, better reflecting usual intake and allowing for mitigation against intra-individual variation. Recalls were grouped six months apart to account for some seasonal variation. Synthetic weeks accounted for differences in diet on weekend days. Race-specific validation was performed, and Black participants were intentionally oversampled. Still, there were a number of limitations. Not all dietary sugars (e.g., lactose) were readily available in the study database. Furthermore, 24-h dietary recalls are not an ideal reference for validity comparisons. In the case of less commonly eaten foods, they may underestimate consumption. They may also be subject to similar recall and reporting biases as FFQs, and thus some of their errors may be correlated. There was also no validation against biomarkers.

Urinary fructose and sucrose from 24-h urine collections have been found to be useful predictor biomarkers at high levels of total and added sugar intake but not at lower intake levels (i.e., high added sugar intake r = 0.77 against urinary biomarker compared to low added sugar intake, r = 0.16) [43]. However, urinary fructose and sucrose from overnight urine samples, as available in AHS-2, have not been established as adequate predictor markers.

## 5. Conclusions

We observed moderate validity for added sugars and fructose and low-moderate validity for total sugars and sucrose measured by the AHS-2 FFQ in this population. Dietary sugar estimates from this FFQ may be useful in assessing possible associations of sugar intakes with health outcomes; the use of calibration methods for such association studies should be considered.

## Figures and Tables

**Table 1:** Participant characteristics for all participants and comparisons of Black and non-Black participants.

	Total		Black		Non-Black		
	(*N* = 904)		(*N* = 376)		(*N* = 507)		
	No.	%	No.	%	No.	%	*p*-Value ^f^
Gender ^b^							0.0869
Males	303	34.3	116	30.9	187	36.9	
Females	580	65.7	260	69.2	320	63.1	
Age (years) ^a^	58.55 ± 13.36		56.05 ± 12.65		60.41 ± 13.57		<0.0001
Dietary pattern ^b^							<0.0001
Vegetarian	521	59.0	191	50.8	330	65.1	
Non-vegetarian	362	41.0	185	49.2	177	34.9	
Body mass index (BMI) ^c,d,e^							<0.0001
<25	351	40.4	118	31.9	233	46.8	
25–29	264	30.4	116	31.4	148	29.7	
≥30	253	29.1	136	36.8	117	23.5	
Mean BMI (kg/m^2^) ^a^	27.28 ± 6.04 ^c^		28.49 ± 6.44 ^d^		26.38 ± 5.56 ^e^		<0.0001

^a^ Values expressed as mean ± standard deviation. ^b^ Missing *n* = 21. ^c^ Missing *n* = 36. ^d^ Missing *n* = 6. ^e^ Missing *n* = 9. ^f^ *p*-value is for the comparison on Black vs. non-Black subjects. Independent sample *t*-test for age, and mean BMI; Pearson chi-squared analysis for gender, dietary pattern, and BMI categories.

**Table 2:** Means, standard deviation, and 10th, 25th, 50th,75th, and 90th percentile distribution for total sugars, added sugars, fructose, and sucrose intake per day assessed by FFQ and multiple 24-h recall (*n* = 904).

Nutrient Intake	24-h						FFQ					
	Mean ± SD	Percentiles					Mean± SD	Percentiles				
		10th	25th	50th Median	75th	90th		10th	25th	50th Median	75th	90th
Total sugars (g)	92.61 ± 40.23	48.48	64.79	86.37	112.71	144.86	103.09 ± 53.65	47.72	67.17	91.55	123.98	171.13
Added sugars (g)	49.51 ± 30.17	19.40	28.92	43.80	62.79	86.62	30.63 ± 25.31	9.90	14.97	24.92	38.47	56.87
Sucrose (g)	39.71 ± 21.02	17.34	25.73	35.64	49.11	65.89	32.67 ± 17.07	14.71	20.45	29.31	40.57	53.53
Fructose (g)	22.84 ± 11.87	9.36	14.39	20.66	29.13	37.48	31.49 ± 20.73	12.51	17.81	27.04	39.05	54.52
Total Energy intake												
Total energy (kcal/day)	1561.31 ± 498.36	953.65	1214.01	1502.58	1840.34	2213.64	1806.23 ± 721.14	993.01	1261.73	1690.26	2215.91	2785.80

Abbreviations: SD, standard deviation, FFQ, food-frequency questionnaire; 24-h, 24-h dietary recall.

**Table 3:** Means and standard deviation for non-energy adjusted total sugars, added sugars, fructose, and sucrose intake per day assessed by FFQ and multiple 24-h recall specified by race (*N* = 883).

	Black (*n* = 376)				Non-Black (*n* = 507)			
	24-h Recall		FFQ		24-h Recall		FFQ	
Nutrient Intake	Mean	SD	Mean	SD	Mean	SD	Mean	SD
Total sugars (g)	84.92	37.44	110.67	64.14	98.67	39.70	96.37	42.13
Added sugars (g)	45.90	29.96	31.07	29.27	52.46	29.32	30.28	22.07
Sucrose (g)	37.12	20.80	34.06	18.69	41.77	20.35	31.34	15.18
Fructose (g)	21.54	11.08	35.98	25.70	23.92	11.95	27.87	14.96
Total Energy intake:								
Total energy (kcal/day)	1448.10	476.99	1812.09	777.65	1656.72	490.99	1783.98	645.15

Abbreviations: SD, standard deviation, FFQ, food-frequency questionnaire, 24-h; 24-h dietary recall.

**Table 4:** Correlations of nutrient intake for FFQ and 24-h recall.

Nutrient	Spearman’s (Non-Energy Adj.)			Pearson’s (Energy Adj.) *		
	All Participants *N* = 904	Black *N* = 376	Non-Black *N* = 507	All Participants *N* = 904	Black *N* = 376	Non-Black *N* = 507
Total sugars	0.20	0.17	0.29	0.34	0.33	0.38
Added sugars	0.31	0.32	0.31	0.34	0.34	0.36
Sucrose	0.17	0.14	0.21	0.24	0.27	0.22
Fructose	0.30	0.29	0.36	0.40	0.36	0.43

Note: * log transformed, abbreviations: adj. = adjusted.

**Table 5:** Uncorrected and deattenuated energy-adjusted correlations of FFQ and 24-h recalls in all subjects and by race.

Nutrient	All		Black		Non-Black	
	Uncorrected *N* = 862	Deattenuated (95% CI) *N* = 862	Uncorrected *N* = 360	Deattenuated (95% CI) *N* = 360	Uncorrected *N* = 483	Deattenuated (95% CI) *N* = 483
Total sugars (g)	0.30	0.40 (0.31–0.50)	0.28	0.36 (0.23–0.48)	0.36	0.51 (0.39–0.64)
Added sugars (g)	0.34	0.48 (0.38–0.58)	0.34	0.46 (0.26–0.59)	0.36	0.52 (0.39–0.65)
Sucrose (g)	0.20	0.32 (0.21–0.42)	0.23	0.35 (0.19–0.49)	0.18	0.30 (0.17–0.44)
Fructose (g)	0.39	0.50 (0.41–0.58)	0.36	0.49 (0.31–0.60)	0.42	0.54 (0.43–0.64)

Abbreviations: CI = confidence interval.

## Supplementary Materials

The following are available online at https://www.mdpi.com/article/10.3390/nu13114152/s1, Figure S1: Scatter plot of total sugars, added sugars, fructose and sucrose intake measured by 24-h recall and FFQ, Table S1: Frequency of occurrence of each written-in value for the “other fruit juice” write-in FFQ question, Table S2: Frequency of occurrence of each written-in value for the “other Canned/Cooked Fruit” write-in FFQ question, Table S3: Frequency of occurrence of each written-in value for the “other Hot drinks” write-in FFQ question, Table S4: Percent agreement between the classification by quintiles of nutrient intake from FFQ and 24-h recall of all study subjects, Table S5: Means and standard deviation for non-energy adjusted total sugars, added sugars, fructose, and sucrose intake per day assessed by FFQ and multiple 24-h recall specified by dietary pattern.
