# Supplementary material for: Validity of FFQ Estimates of Total Sugars, Added Sugars, Sucrose and Fructose Compared to Repeated 24-h Recalls in Adventist Health Study-2 Participants
Source: Nutrients. 2021 Nov 19;13(11):4152. doi: 10.3390/nu13114152 (PMC8622229; doi:10.3390/nu13114152)
Supplement: Supplementary file 1 [file nutrients-13-04152-s001.zip › nutrients-1460190-supplementary.pdf]

Supplemental

Figure S1. Scatter plot of total sugars, added sugars, fructose and sucrose intake measured by 24-hour recall and FFQ

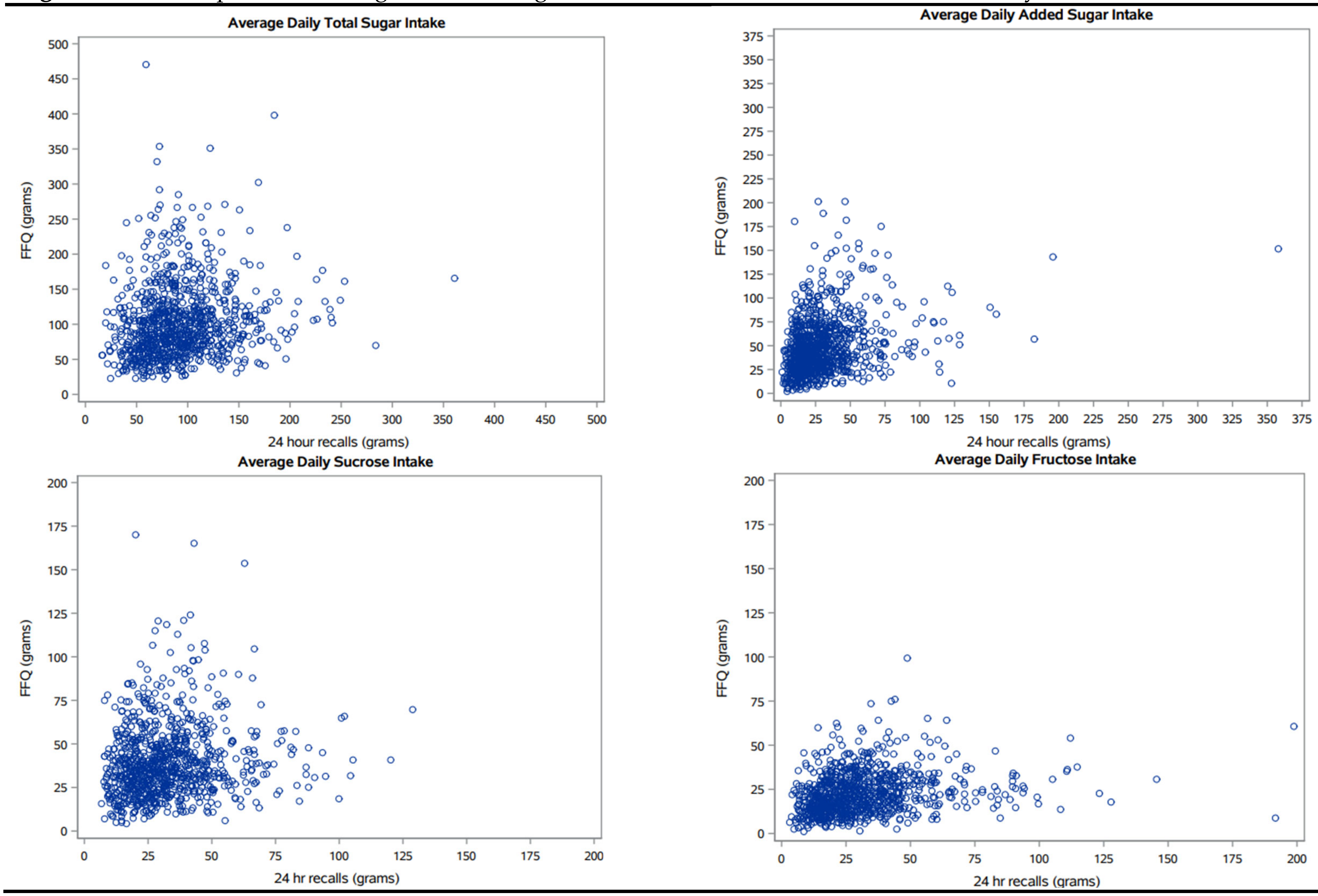

Supplemental

Table S1: Frequency of occurrence of each written-in value for the "other fruit juice" write-in FFQ question. Bolded percentages exceed the 3% occurrence minimum for each intake frequency; the corresponding juice was thus included as a component in the final weighted meta-recipe for that intake frequency.

Meta-Recipe Components for Fruit Juice	1-3/month	1/week	2-4/week	5-6/week	1/day	2-3/day	4-5/day
Cranberry drink or cocktail	**32%**	**30%**	**31%**	**30%**	**31%**	**35%**	n/a
Grape juice	**29%**	**31%**	**34%**	**30%**	**32%**	**24%**	n/a
Grapefruit juice	**8%**	**8%**	**9%**	**11%**	**10%**	**8%**	n/a
Lemonade	1%	2%	2%	2%	1%	**4%**	n/a
Mixed Juice	**17%**	**19%**	**17%**	**22%**	**22%**	**25%**	n/a
Pineapple juice	**12%**	**10%**	**7%**	**6%**	**5%**	**4%**	n/a

Table S2: Frequency of occurrence of each written-in value for the "other Canned/Cooked Fruit" write-in FFQ question. Bolded percentages exceed the 3% occurrence minimum for each intake frequency; the corresponding canned/cooked fruit was thus included as a component in the final weighted meta-recipe for that intake frequency.

Meta-Recipe Components for Canned/Cooked Fruit	1-3/month	1/week	2-4/week	5-6/week	1/day	2-3/day	4-5/day
Apple or applesauce	**13%**	**22%**	**24%**	**36%**	**39%**	n/a	n/a
Fruit cocktail	**7%**	**5%**	**6%**	**5%**	**8%**	n/a	n/a
Peach	**51%**	**47%**	**49%**	**42%**	**28%**	n/a	n/a
Pear	**4%**	**5%**	**4%**	2%	2%	n/a	n/a
Pineapple	**13%**	**10%**	**6%**	**6%**	**7%**	n/a	n/a
Fried plantains	**5%**	**5%**	**6%**	**5%**	**8%**	n/a	n/a
Boiled or baked plantains	**5%**	**5%**	**6%**	**5%**	**8%**	n/a	n/a

Table S3: Frequency of occurrence of each written-in value for the "other Hot drinks" write-in FFQ question. Bolded percentages exceed the 3% occurrence minimum for each intake frequency; the corresponding hot drink was thus included as a component in the final weighted meta-recipe for that intake frequency.

Meta-Recipe Components for Hot drinks	1-3/month	1/week	2-4/week	5-6/week	1/day	2-3/day	4-5/day
Hot chocolate milk mixture	**27%**	**21%**	**16%**	**11%**	**8%**	**4%**	2%
Coffee, cappuccino	1%	1%	1%	1%	**3%**	2%	2%
Coffee substitute	**26%**	**27%**	**29%**	**32%**	**35%**	**20%**	**27%**
Green tea	**24%**	**26%**	**31%**	**32%**	**36%**	**48%**	**52%**

Herbal tea	**22%**	**25%**	**24%**	**24%**	**19%**	**26%**	**17%**

Table S4: Percent agreement between the classification by quintiles of nutrient intake from FFQ and 24-hr recall of all study subjects

	**Non-energy adj.**				**Energy adj.**			
	**Exact agreement**	**Adjacent agreement**	**Exact and adjacent combined**	**Gross misclassification**	**Exact agreement**	**Adjacent agreement**	**Exact and adjacent combined**	**Gross misclassification**
All:								
Total sugar	23.3%	33.4%	56.7%	5.4%	28.3%	35.3%	63.6%	4.1%
Added sugar	28.5%	32.9%	61.5%	4.6%	28.4%	34.9%	63.3%	4.2%
Sucrose	23.1%	36.4%	59.5%	6.7%	24.8%	36.0%	60.8%	4.5%
Fructose	26.7%	33.9%	60.6%	3.1%	31.8%	33.5%	65.3%	2.3%
Black study subjects:								
Total sugar	24.5%	34.6%	59.0%	5.3%	29.5%	32.2%	61.7%	4.3%
Added sugar	29.0%	32.4%	61.4%	4.3%	27.9%	35.4%	63.3%	4.3%
Sucrose	22.3%	38.0%	60.4%	6.9%	29.0%	30.9%	59.8%	4.5%
Fructose	25.5%	37.2%	62.8%	3.7%	25.8%	37.8%	63.6%	2.4%
Non-Black study subjects:								
Total sugar	25.8%	34.7%	60.6%	3.4%	27.2%	39.3%	66.5%	3.7%
Added sugar	31.0%	31.0%	61.9%	3.6%	30.8%	33.3%	64.1%	3.6%
Sucrose	28.0%	32.1%	60.2%	4.7%	22.5%	37.3%	59.8%	4.7%
Fructose	26.6%	37.3%	63.9%	2.6%	34.9%	31.6%	66.5%	2.6%

Note: adj.=Adjusted. Percent agreement based on classification into quintiles. Exact agreement is the proportion of subjects classified into the same quintile. Adjacent agreement is the percent of subjects that were classified into ±1 quintile from exact. Gross misclassification represents the percentage of participants with FFQ and 24-hour recall intakes that grossly misclassified in the extreme quintiles.

Table S5. Means and Standard Deviation for Non-energy Adjusted Total Sugars, Added Sugars, Fructose, and Sucrose Intake Per Day Assessed by FFQ and Multiple 24-hour Recall Specified by Vegetarian and Non-Vegetarian Diet (N=883)

	**Vegetarian (n=521)**				**Non-Vegetarian (n=362)**			
	**24- hour recall**		**FFQ**		**24- hour recall**		**FFQ**	
Nutrient intake:	Mean	SD	Mean	SD	Mean	SD	Mean	SD
Total sugars (g)	96.07	40.21	101.92	50.80	88.13	37.59	103.23	56.26
Added sugars (g)	49.91	30.40	28.12	20.72	49.32	28.84	34.21	30.54
Sucrose (g)	40.91	20.61	32.70	15.81	38.17	20.65	32.20	18.18
Fructose (g)	24.39	11.73	31.61	19.50	20.77	11.19	30.91	22.16
Total Energy intake:								
Total energy (kcal/d)	1589.76	510.89	1805.44	690.73	1536.41	471.83	1782.29	724.22

Abbreviations: SD, Standard Deviation, FFQ, food-frequency questionnaire, 24-HR; 24- hour dietary recall
